# DNA Interaction and DNA Cleavage Studies of a New Platinum(II) Complex Containing Aliphatic and Aromatic Dinitrogen Ligands

**DOI:** 10.1155/2011/525794

**Published:** 2011-12-22

**Authors:** Nahid Shahabadi, Soheila Kashanian, Maryam Mahdavi, Noorkaram Sourinejad

**Affiliations:** Department of Chemistry, Faculty of Science, Razi University, Kermanshah 74155, Iran

## Abstract

A new Pt(II) complex, [Pt(DIP)(LL)](NO_3_)_2_ (in which DIP is 4,7-diphenyl-1,10-phenanthroline and LL is the aliphatic dinitrogen ligand, *N,N*-dimethyl-trimethylenediamine), was synthesized and characterized using different physico-chemical methods. The interaction of this complex with calf thymus DNA (CT-DNA) was investigated by absorption, emission, circular dichroism (CD), and viscosity measurements. The complex binds to CT-DNA in an intercalative mode. The calculated binding constant, *K_b_*, was 6.6 × 10^4^ M^−1^. The enthalpy and entropy changes of the reaction between the complex and CT-DNA showed that the van der Waals interactions and hydrogen bonds are the main forces in the interaction with CT-DNA. In addition, CD study showed that phenanthroline ligand insert between the base pair stack of double helical structure of DNA. It is remarkable that this complex has the ability to cleave the supercoiled plasmid.

## 1. Introduction

Cisplatin is one of the most potent antitumor drugs available for the therapeutic management of solid tumors, such as germ cell tumors, ovarian, lung, head and neck, and bladder cancers, and so forth. Despite its wide application as a chemotherapeutic agent, cisplatin exhibits severe side effects, such as nephrotoxicity, neurotoxicity, ototoxicity, nausea, and emetogenicity, which limits the possibilities for gaining therapeutic benefits from dose intensification [1–3]. Thus, a plethora of Pt(II) and Pt(IV) complexes with nitrogen-containing ligands has been the subject of intensive biological evaluation aimed at developing less toxic and more selective anticancer therapeutics [4, 5]. The DNA-binding mechanism and behavior of the complexes are closely related to the size, shape, and planarity of the intercalative ligands. Besides the above-mentioned factors, ancillary ligands play an important role in DNA-binding behaviors of complexes. Propanediamine derivatives of the type [PtCl_2_(N-benzyl-1,3-propanediamine)_2_] have been reported as potential antitumor agents [6]. In addition, the metal complexes bound to DNA through the noncovalent interaction generally form an important subgroup, of which the members are cations and typically contain ligands bearing extended hydrophobic areas or surfaces [7]. In recent years, polypyridyl complexes that can recognize and bind at specific DNA sites have received considerable attention [8]. Many efforts have been directed toward the design of complexes containing modified bipy or phen ligands that bind DNA primarily via base-pair intercalation [9]. The examples of this category of DNA-binding agents are metal complexes with polypyridines or 1,10-phenanthrolines (phen) and their derivatives such as 4,7-diphenyl-1,10-phen (DIP) and dipyrido [3,2-a; 29,39-c]phenazine (dppz) [7].

The application of mixed-ligand complexes permits variation in the geometry, size, and hydrophobicity by systematically adjusting the ligands and their substituents and allows the opportunity to determine how these various factors contribute to the affinity in DNA binding. By systemically comparing binding constants and thermodynamic parameters for a mixed-ligand complex, we may determine the contributions of the different ligand functionalities and sizes to the DNA-binding characteristics [10]. The majority of research in this field has focused on the use of polypyridine derivatives such as 2,2′-bipyridine and 1,10-phenanthroline as ligands [11–14].

Therefore, to design improved drugs that target DNA and to investigate the effect of mixed-ligand complexes on the structure and conformation of DNA, we used a Pt(II) complex, [Pt(DIP)(LL)](NO_3_)_2_ (in which DIP is 4,7-diphenyl-1,10-phenanthroline and LL is the aliphatic dinitrogen ligand, *N,N*-dimethyl-trimethylenediamine). Binding studies of this complex with calf thymus DNA (CT-DNA) were studied by electronic absorption spectroscopy, fluorescence spectroscopy, circular dichroic spectral, viscosity measurements, and electrophoresis.

## 2. Experimental

### 2.1. Materials

4,7-diphenyl-1,10-phenanthroline (DIP), *N,N*-dimethyltrimethylenediamine, hydrazine dihydrochloride, potassium Chloride, nitric acid, Tris-acetate-EDTA (TAE) buffer, hydrogen peroxide, NaN_3_, and Tris-HCl were purchased from Merck. Doubly distilled deionized water was used throughout. Highly polymerized CT-DNA and pUC19 DNA was also purchased from Sigma and used without purification.

 Experiments were carried out in Tris-HCl buffer at pH 7.2. A solution of calf thymus DNA gave a ratio of UV absorbance at 260 and 280 nm more than 1.8, indicating that DNA was sufficiently free from protein. The stock solution of CT-DNA was prepared by dissolving of DNA in 10 mM of the Tris-HCl buffer at pH 7.2. The DNA concentration (monomer units) of the stock solution (1 × 10^−2 ^M per nucleotide) was determined by UV spectrophotometer, in properly diluted samples, using the molar absorption coefficient 6600 M^−1 ^cm^−1^ at 258 nm [15]. The stock solutions were stored at 4°C and used over no more than 4 days.

### 2.2. Synthesis of Platinum Complex

#### 2.2.1. Synthesis of Pt(DIP)Cl_2_ [16]

Pt(DIP)Cl_2_ complex (DIP = chelating diamin ligand: 4,7-diphenyl, 1,10-phenanthroline) were synthesized by boiling of K_2_PtCl_4_ (1.0 g) and DIP ligand (0.5 g) in 1.5 mL of water containing 0.5 mL of HCl. After 20 h, the reaction mixture was allowed to cool to room temperature and the light green product that precipitated was filtered out, washed with hot water and dried under vacuum. Yield: 1.04 g (97%).

#### 2.2.2. Synthesis of [Pt(DIP)(LL)](NO_3_)_2_

Pt(DIP)Cl_2_ (0.08 g, 0.167 mmol) was dissolved in 2 mL of DMF. A solution of silver nitrate (0.056 g, 0.333 mmol) in 1 mL of DMF was added and the mixture was stirred overnight at room temperature in the dark. The precipitate was filtered off and LL ligand (LL = N,N-dimethyltrimethylenediamine) (0.017 g, 0.167 mmol) was added to the filtrate. The mixture was stirred for 4 days at room temperature in the dark. The product was washed with a small amount of ethanol and dried under vacuum. Yield: 0.041 g (47%). ^1^H NMR (ppm, CDCl_3_): 7.48(Ph); H^1′^ (9.58); H^2′^ (8.07); H^3′^ (8.01); ^1^CH_2_(3.03); ^2^CH_2_(1.86); ^3^CH_2_(2.46); NH_2_(2.16); Me(2.41). Anal. Calc. for PtC_29_N_4_ H_30_: C, 55.32; H, 4.80; N, 8.90; Found: C, 55.11; H, 5.1; N, 8.69%. FT-IR data (Cm^−1^): 3309, 2935, 1664, 1577, 1439, 548, 420.

### 2.3. Instrumentation

^1^H NMR spectra were recorded using a Bruker Avance DPX200 MHz (4.7 Tesla) spectrometer with CDCl_3_ as the solvent. The elemental analysis was performed using a Heraeus CHN elemental analyzer. The molar conductance of complexes was measured in DMF at room temperature on an ELICO (CM 82T) conductivity bridge.

Absorbance spectra were recorded using an HP spectrophotometer (Agilent 8453) equipped with a thermostated bath (Huber polysat cc1). Absorption titration experiments were conducted by keeping the concentration of complexes constant (5 × 10^−5^ M), while varying the DNA concentration from 0 to 1 × 10^−4^ M (*r*_*i*_ = [DNA]/[complex] = 0.0,0.1,0.6,1, 1.5, 2). Absorbance values were recorded after each successive addition of DNA solution, followed by an equilibration period. In order to obtain a more quantitative determination of the interaction strength, intrinsic-binding constant, *K*_*b*_, was determined using spectroscopic titration data at 230 nm. The data were then fitted to (1) to obtain the binding constant [17],

(1) $$\frac{\left[\text{DNA}\right]}{\left(\varepsilon_{a}-\varepsilon_{f}\right)}=\frac{\left[\text{DNA}\right]}{\left(\varepsilon_{b}-\varepsilon_{f}\right)\;}+\frac{1}{K_{b}\left(\varepsilon_{b}-\varepsilon_{f}\right)},$$

where *ε*_*a*_, *ε*_*f*_, and *ε*_*b*_, are the apparent, free, and bound metal complex extinction coefficients, respectively. In particular, *ε*_*f*_  was determined by a calibration curve of the isolated metal complex in aqueous solution, following the Beer's law. *ε*_*a*_ was determined as the ratio between the measured absorbance and the Pt(II) complex concentration, *A*_obs_/[complex]. A plot of [DNA]/(*ε*_*b*_ − *ε*_*f*_) versus [DNA] gave a slope of +1/(*ε*_*b*_ − *ε*_*f*_) and a *Y* intercept equal to 1/*K*_*b*_(*ε*_*b*_ − *ε*_*f*_); *K*_*b*_ is the ratio of the slope to the *Y* intercept.

Viscosity measurements were made using a viscosimeter (SCHOT AVS 450) that was maintained at 25 ± 0.5°C using a constant temperature bath. The DNA concentration was fixed at 5 × 10^-5 ^M, and flow time was measured with a digital stopwatch. The mean values of three replicated measurements were used to evaluate the viscosity *η* of the samples. The values for relative specific viscosity (*η*/*η*_o_)^1/3^, where *η*_o_ and *η* are the specific viscosity contributions of DNA in the absence (*η*_o_), and in the presence of the Pt (II) complex (*η*) were plotted against *r*_*i*_ (*r*_*i*_ = [complex]/[DNA] = 0.0,0.1,0.3,0.6,0.9,1.2) [18].

Fluorescence measurements were carried out with a JASCO spectrofluorimeter (FP 6200) by keeping the concentration of complex constant (5 × 10^-5 ^M) while varying the DNA concentration from 0 to 6 × 10^-5 ^M. (*r*_*i*_ = [DNA]/[complex] = 0.0, 0.4, 0.6, 0.8, 1, 1.2) at three different temperatures (283, 288, 310 K). Stern-Volmer Constant (*K*_sv_) is used to evaluate the fluorescence quenching efficiency. According to the classical Stern-Volmer equation:

(2) $$\frac{F_{0}}{F}=1+K_{q}\tau_{0}\left[Q\right]=1+K_{\text{sv}}\left[Q\right],$$

where *F*_0_ and *F* represent the fluorescence intensities in the absence and in the presence of quencher, respectively. *K*_*q*_ is the quenching rate constant of biomolecule,  *K*_sv_ the dynamic quenching constant, *τ*_0_ is the lifetime of the biomolecule without quencher (*τ*_0_  = 10^−8^), and [*Q*] is the concentration of quencher.

CD measurements were recorded on a JASCO (J-810) spectropolarimeter by keeping the concentration of DNA constant (5 × 10^-5 ^M) while varying the complex concentration from 0 to 4 × 10^-5 ^M (*r*_*i*_ = [complex]/[DNA] = 0,0.2,0.4,0.6,0.8).

### 2.4. Cleavage Efficiency

The DNA cleavage activity of the metal complex was studied by using agarose gel electrophoresis. Supercoiled plasmid pUC19 DNA (50 *μ*mol) was dissolved in a 0.050 mol Tris-(hydroxymethyl) methane-HCl (Tris-HCl) buffer (pH 7.2) containing 0.050 mol NaCl and the different concentration of complexes (100, 200, 300, and 400 *μ*mol). The mixtures were incubated at 37°C for 24 h and then mixed with the loading buffer (2 *μ*L) containing 25% bromophenol blue, 0.25% xylene cyanol, and 30% glycerol. Each sample (5 *μ*L) was loaded into 0.8% w/v agarose gel. Electrophoresis was undertaken for 1 h at 50 V in Tris-acetate-EDTA (TAE) buffer. The gel was stained with ethidium bromide for 5 min after electrophoresis, and then photographed under UV light. The proportion of DNA in each fraction was quantitatively estimated from the intensity of each band with the Alpha Innotech Gel documentation system (AlphaImager 2200). To enhance the DNA-cleaving ability by the complexes, hydrogen peroxide (100 *μ*mol) was added into each complex (400 *μ*mol). Moreover, the cleavage mechanism was further investigated by using scavengers for the hydroxyl radical species (4 *μ*L, DMSO) and the singlet oxygen species (100 *μ*mol, NaN_3_). All experiments were carried out in triplicate under the same conditions.

## 3. Results and Discussions

### 3.1. Synthesis and Characterization of Platinum Complex

**Scheme 1:**
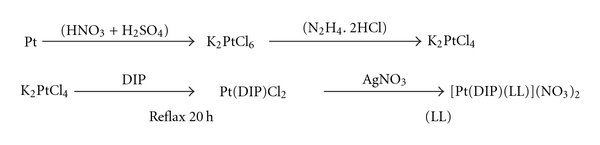


The platinum complex, [Pt(DIP)(LL)](NO_3_)_2_ in which LL = *N,N*-dimethyltrimethylenediamine and DIP = 4,7-diphenyl-1,10-phenanthroline, was synthesized from reaction of K_2_PtCl_4_ and diimine ligands (Scheme 1) and characterized by UV-Vis, IR and NMR spectroscopic methods and elemental analysis.

**Figure 1:**
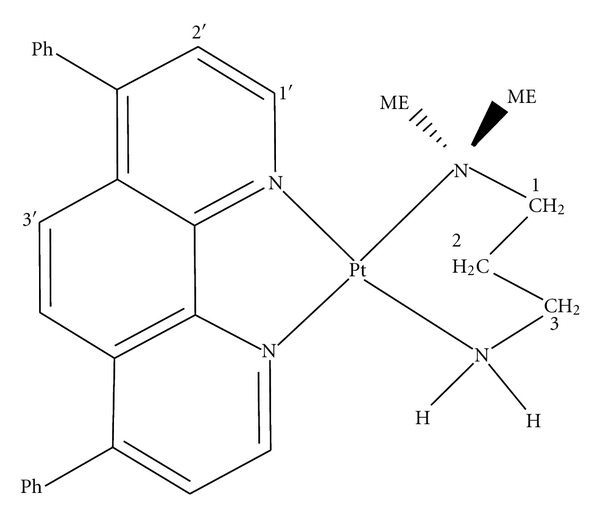
The structure and ^1^H NMR labeling of the [Pt(DIP)(LL)](NO_3_)_2_ complex.

**Figure 2:**
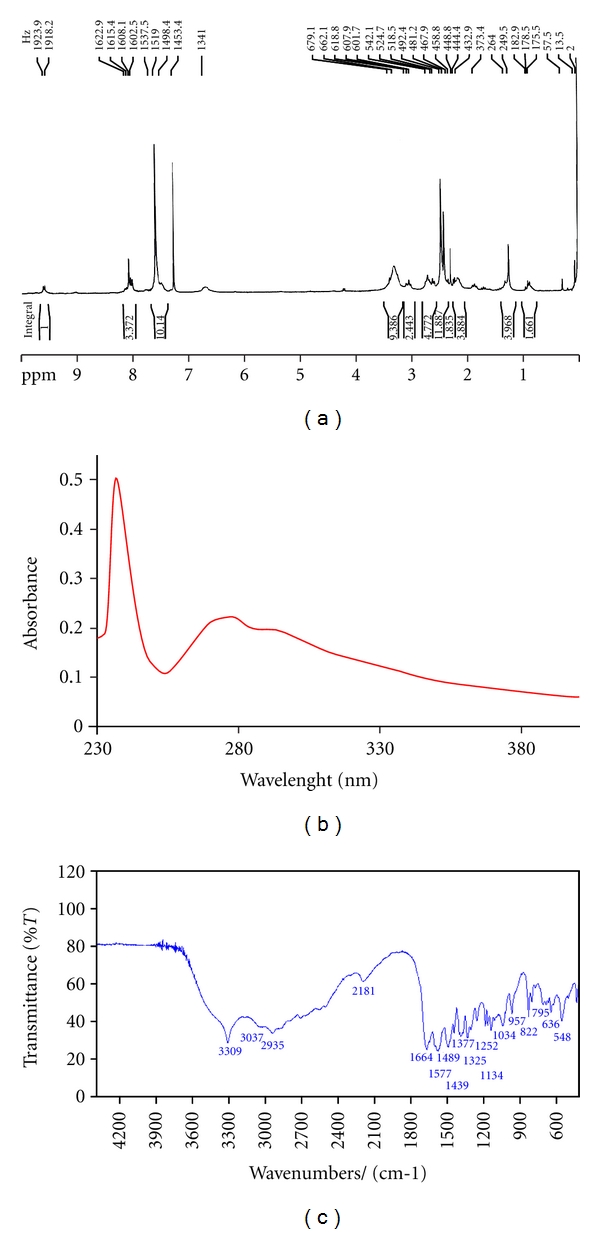
(a) ^1^H NMR spectrum, (b) UV-vis spectrum, and (c) FT-IR spectrum of the platinum complex.

The ^1^H NMR Labeling is shown in Figure 1. The ^1^H NMR spectrum (Figure 2(a)) has shown proton signals at aliphatic (0–4 ppm) and aromatic (7–10 ppm) regions. The ring current of 4,7-diphenyl-1,10-phenanthroline (DIP) ligand with shielding due to nonbonded interaction of protons with metal causes a complex resonance pattern at low field. The feature specially led to some coupling of alpha protons with ^195^Pt and so that signal shift to higher field. Therefore, the set of signals of aromatic and aliphatic ligands shift to higher field this is characteristic for attaching the ligand to platinum metal. The unsymmetrical structure of the platinum complex causes that the chemically equivalent protons of phenanthroline ligand resonance at different chemical shifts. ^1^H NMR chemical shifts of the free ligands and platinum complex are shown in Table 1. The absorption spectrum of the Pt(II) complex is shown in Figure 2(b). In the UV region, the complex exhibits two intense absorption bands around 237 and 280 nm which is attributed to *n* → π* and π → π* transitions of dinitrogen ligands. The MLCT band is observed in visible region around 430 nm. Therefore, the absorption spectrum confirms the suggested structure for the platinum complex. The FT-IR spectrum of the Pt(II) complex is shown in Figure 2(c). The coordination of the nitrogen atoms is confirmed with the presence of two bands at 548 and 420 cm^−1^, assignable to *ν*(Pt-N) for aliphatic and aromatic ligands, receptively. Furthermore, the appearance of two bands around 1577–1664 cm^−1^ is indicative of bending vibration of N–H groups. The broad band of N–H stretching vibration is also observed at 3309 cm^−1^. The bands around 1439 and 1577 cm^−1^ can be attributed to the ring stretching frequencies [*ν* (C=C) and *ν* (C=N)] of phenanthroline ligand. The bands around 2935 cm^−1^ can be assigned to C–C stretching vibration of aliphatic CH_2_ groups of aliphatic ligand.

### 3.2. Electronic Absorption Spectral Studies

**Figure 3:**
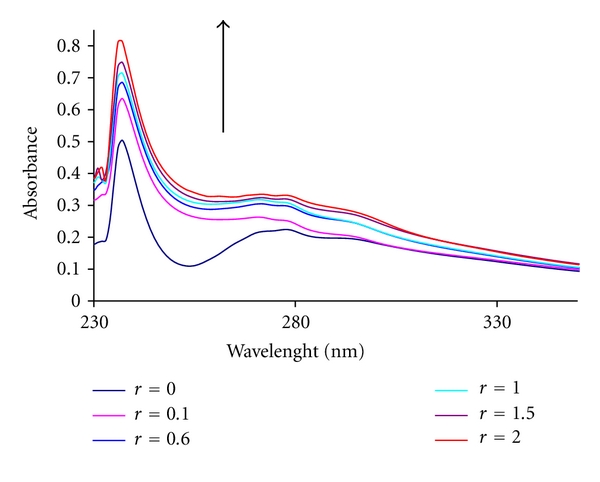
Absorption spectra of Pt(II) complex (5 × 10^−5^ M) in the absence and presence of increasing amounts of CT DNA: *r*_*i*_ = 0.0, 0.1, 0.6, 1. 1.5, and 2.

In general, hypochromism and redshift are associated with the binding of the complex to the helix by an intercalative mode involving strong stacking interaction of the aromatic chromophore of the complex between the DNA base pairs.  Figure 3 shows the UV absorption spectra of Pt(II) complex in the absence and presence of CT-DNA. The absorption intensity of the complex increased (hyperchromism) evidently after the addition of DNA, which indicated the interactions between DNA and the complex.

The Pt(II) complex can be bind to double-stranded DNA in different binding modes on the basis of the structure and type of the ligands. As DNA-double helix possesses many hydrogen binding sites which are accessible both in the minor and major grooves, it is likely that the amine group of aliphatic ligand in the Pt(II) complex forms hydrogen bands with DNA, which may contribute to the hyperchromism observed in the absorption spectra. On the other hand, our platinum complex, which possesses methylene groups of the aliphatic diamine ligand (LL) can be bind to DNA by van der Waals interaction between the methylene groups and the thymine methyl group [19]. The hyperchromic effect may also be due to the electrostatic interaction between positively charged cation and the negatively charged phosphate backbone at the periphery of the double helix-CT-DNA [20]. In the titled complex, the complete intercalation of phenanthroline ligands between set of adjacent base pairs is sterically impossible, but some type of partial intercalation can be envisioned [21]. But this needs further clarification of the DNA-binding mode of the complex by viscosity measurements.

The calculated *K*_*b*_ value was found to 6.6 × 10^4^ M^−1^. The values of *K*_*b*_ described in the literature for classical intercalators (ethidium-DNA, 7 × 10^7^ M^−1^), [22] (proflavin-DNA, (4.1 × 10^5^ M^−1^) [23] are at least ten order in magnitude higher than that of this Pt(II) complex. This result suggests that intercalation between base pairs is not the main mode of interaction of Pt(II) complex with DNA. In contrast, the value of *K*_*b*_ is ten order in magnitude higher than *K*_*b*_ values which found for compounds with the mode of groove binding to DNA like Cr(III) complexes [24] or Tris (1,10-phen) ruthenium (II) to DNA [25]. Theses results confirm that the platinum complex strongly interact with DNA, the *K*_*b*_ value of this complex is similar to ZnL^2+^ complex in analogue condition (*K*_*b*_ = 7.35 × 10^4^ M^−1^) which considered as an intercalating complex [26].

Furthermore, the *K*_*b*_ value obtained for our complex is lower than some platinum complexes which suggested as intercalators and have similar structures such as [Pt(en) (5,6-Me_2_-Phen)]Cl_2_ (*K*_*b*_ = 1.5 × 10^6^); [Pt(en)(3,4,7,8-Me_4_-Phen)]Cl_2_ (*K*_*b*_ = 7 × 10^5^) [27].

From the results, we can deduce that the platinum complex bind to DNA by intercalation and the weaker DNA binding of the complex may arise from the steric effect of the phenyl groups on phenanthroline ligands which hinder the complete insertion of DIP ligand between the DNA base pairs. This type of steric clash has been also suggested for the binding of [Ru(5,6-dmp)(NH_3_)_4_]^2+^ [28] and [{5,6-dmp)_2_Ru}_2_(bmp)]^4+^ [29].

Therefore, we suggest that the present platinum complex bind to DNA most probably through intercalation, but to further clarification of the DNA-binding mode, viscosity measurements should be done.

### 3.3. Viscosity Studies

**Figure 4:**
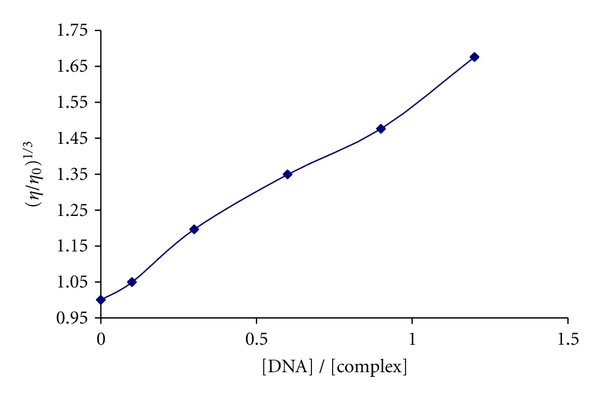
Effect of increasing amounts of complex on the viscosity of CT-DNA (5 × 10^−5^ M) in 10 mM Tris-HCl.

It is known that the classical organic intercalator, Ethidium bromide, increases the axial length of the DNA and it becomes more rigid, resulting in an increase in the relative viscosity. Results confirm the sensitivity of viscosity measurements to the different modes of DNA binding. The values of relative specific viscosity (*η*/*η*∘)^1/3^ (*η*∘ and *η* are the specific viscosity contributions of DNA in the absence and in the presence of the present complex) were plotted against 1/*R* (*R* = [DNA]/[complex]) (Figure 4). In the viscosity curve the results indicate that the absence and the presence of the metal complex have a marked effect on the viscosity of the DNA. The specific viscosity of the DNA sample increases obviously with the addition of the complex. The viscosity studies provide a strong argument for intercalation [30, 31]. The relative viscosity of DNA increases with increase in the concentration of Pt(II) complex which is ascribed to the intercalative binding mode of the complex because this could cause the effective length of the DNA to increase [32, 33]. In essence, the length of the linear piece of B-form DNA is given by the thickness of the base pairs that are stacked along the helix axis in van der Waals contact with each other introducing another aromatic molecule into the stack, therefore, the increase of the DNA caused by addition of the complex can provide further support for intercalative mode of the platinum complex binding. It was observed that increasing the platinum complex concentration led to an increase of the DNA viscosity. Thus, we may deduce that the mentioned complex can be considered as a DNA intercalator.

Li et al. [34] showed that EB increased the relative viscosity of DNA and the slope of the graph of (*η*/*η*∘)^1/3^ versus 1/*R* was 0.96, which is very close to value of 1.0 predicted from the theory of Satyanarayana et al. [35]. Since the intercalative interaction of this Pt(II) complex with DNA can make DNA longer, we would expect that the relative viscosity of DNA increases with a slope between 0 and 0.96 (a value measured for EB) if the intercalation of the Pt(II) complex was either only one interaction mode or much stronger than other interaction(s). But in this study the relative viscosity of DNA increase with a slope of 0.46 and it is reasonably believed that may be other interaction(s) between DNA and the Pt(II) complex occurred and is responsible for the decrease of the slope. In addition, the greater increase in viscosity observed for EB compared to the Pt(II) complex is likely due to the lower binding constant of the latter to DNA. These results clearly show the importance of using several techniques to ascertain intercalation. 

### 3.4. Fluorescence Studies

Fluorescence quenching can occur by different mechanisms, which are usually classified as dynamic quenching and static quenching. In general, dynamic and static quenching can be distinguished by their differing dependence on temperature and excited state lifetime [36, 37].

**Figure 5:**
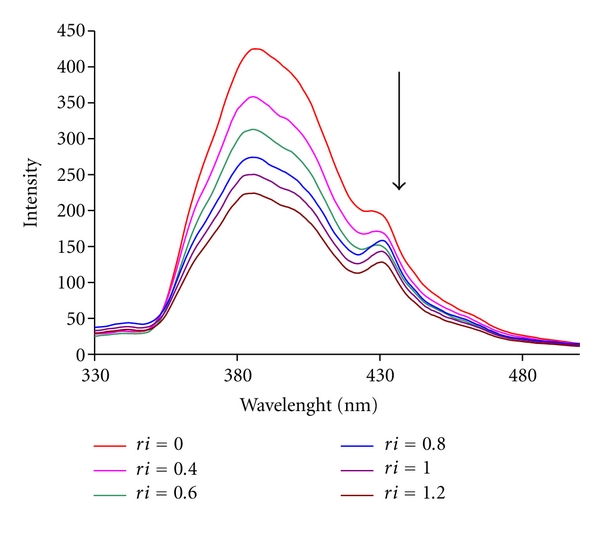
Fluorescence spectra of Pt(II) complex in the presence of various concentrations of CT DNA.

**Figure 6:**
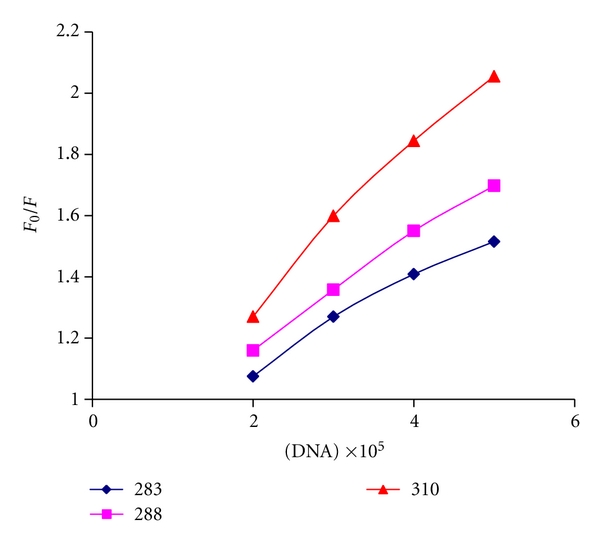
Plots of *F*_0_/*F* versus [*Q*] for the binding of Pt(II) complex with CT DNA.

The effect of DNA on Pt(II) complex fluorescence intensity is shown in Figure 5. As shown in this figure, upon the addition of CT-DNA, an obvious decrease in emission intensity was observed for the complex. This implies that the titled complex has an interaction with DNA. Furthermore, the quenching of luminescence of Pt(II) complex by CT-DNA may be attributed to the photoelectron transfer from guanine base of DNA to the excited MLCT state, as reported in the case of some complexes [38–40]. The plots of *F*_0_/*F* versus DNA at different temperatures are shown in Figure 6. As mentioned in the literature, dynamic quenching or collisional quenching requires contact between the excited flourophore and the quenching specie, the quencher. The other form of quenching is static quenching in which the quencher and the fluorophore in ground state form a stable complex. Fluorescence is only observed from the unbound fluorophore [41].

By using (2), the *K*_sv_ of Pt(II) complex formation by DNA at different temperatures (283, 288, and 310 K) was obtained and the results are shown in Table 2. These results show that the probable quenching mechanism of Pt(II) complex formation by DNA is a dynamic quenching procedure, because the *K*_sv_ has been increased by temperature rising [42].

#### 3.4.1. Binding Constants and Binding Sites

The binding constant (*K*_*f*_) and the binding stoichiometry (*n*) for the complex formation between Pt(II) complex and DNA was measured using the following equation [43]:

(3) $$\frac{\log\left(F_{0}-F\right)}{F}=\log K_{f}+n\;\log\left[\text{DNA}\right].$$

Here *F*_0_ and *F* are the fluorescence intensities of the fluorophore in the absence and in the presence of different concentrations of DNA, respectively. The linear equations of  log⁡(*F* − *F*_0_)/*F* versus log[DNA] at different temperature are shown in Table 3. The values of *K*_*f*_  clearly underscore the affinity of Pt(II) complex to DNA.

#### 3.4.2. Thermodynamic Studies

The interaction forces between drug and biomolecule may involve hydrophobic forces, electrostatic interactions, van der Waals interactions, hydrogen bonds, and so forth [44, 45]. According to the data of enthalpy changes (Δ*H*) and entropy changes (Δ*S*), the model of interaction between drug and biomolecule can be concluded [46]: (1) Δ*H* > 0 and Δ*S* > 0, hydrophobic forces; (2) Δ*H* < 0 and Δ*S* < 0, vander Waals interactions and hydrogen bonds; (3) Δ*H* < 0 and Δ*S* > 0, electrostatic interactions [47]. In order to elucidate the interaction of Pt(II) complex with DNA, the thermodynamic parameters were calculated. The plot of ln⁡*K* versus 1/*T* (4) allows the determination of enthalpy change (Δ*H*) and entropy change (Δ*S*). If the temperature does not vary significantly, the enthalpy change (Δ*H*) can be regarded as a constant. Based on the binding constants at different temperatures, the free energy change (Δ*G*) can be estimated (Table 3; (5)) by the following equations:

(4) $$\begin{array}{c} \ln K=-\frac{\Delta H}{RT}+\frac{\Delta S}{R},\\ \Delta G=\Delta H-T\Delta S=-RT\ln K, \end{array}$$

where *K* is the Stern-volmer quenching constant at the corresponding temperatures and *R* is the gas constant. When we apply this analysis to the binding system of Pt(II) complex and CT-DNA, we find that Δ*H* < 0 and Δ*S* < 0. Therefore, van der Waals interactions or hydrogen bonds are the main forces in the binding of the investigated Pt(II) complex to CT-DNA, and the mode of binding is intercalation. In addition, the negative entropy change results from the intercalation of Pt(II) complex between CT-DNA bases, accompanied by the loss of translational and rotational degrees of freedom.

### 3.5. Circular Dichroic Spectral Studies

**Figure 7:**
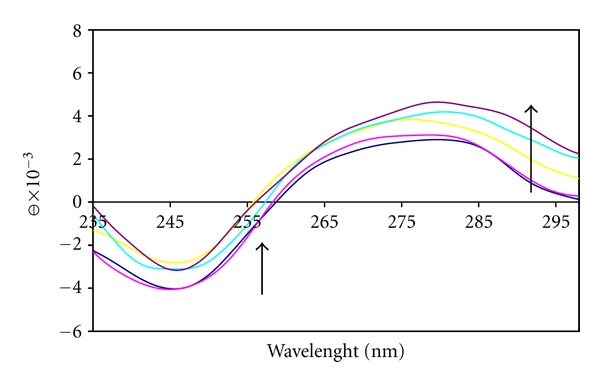
Circular dichroism spectra of CT DNA (5 × 10^−5^ M) in Tris-HCl (10 mM), in the presence of increasing amounts of the complex at the following stoichiometric ratios: *r*_*i*_ = [complex]/[DNA] = 0.0, 0.2, 0.4, 0.6, and 0.8.

The CD spectral technique is useful in monitoring the conformational variations of DNA in solution. The observed CD spectrum of natural DNA consists of a positive band at 275 nm due to base stacking and a negative band at 245 nm due to helicity, which is characteristic of DNA in right-handed B form (Figure 7) [48].

The effect of [Pt(DIP)(LL)](NO_3_)_2_ complex on the conformation of secondary structure of CT-DNA was studied by keeping the concentration of CT-DNA at 5 × 10^-5 ^M^−1^ while varying the concentration of platinum complex in a buffer solution of 10 mM Tris was added, (*r*_*i*_ = 0.2,0.4,0.6,0.8). In the presence of the complex both positive and negative peaks of CD spectra of DNA increased (Figure 7). The changes in ellipticity and wavelength caused by platinum complex is significant and addition of the complex to DNA solution results, both before and after illumination, in a significant and dose-dependent increase of the positive and negative dichroic bands accompanied by a shift to lower energy (redshift). This behavior could be attributed to the insertion of phenanthroline ligand between the base-pair stack of double-helical structure of the DNA. Moreover, the observed increase of the hyperchromicity as well as of the ellipticity following illumination shows the promise of our platinum complex as a photodynamic therapy agent. Thus, further studies to verify the photo-cleavage of DNA in the presence of oxygen should be done.

### 3.6. DNA Cleavage Activity

The degree to which the platinum complex could function as DNA cleavage agent was examined using supercoiled pUC19 plasmid DNA as the target. The efficiency of cleavage of the molecule was probed using agarose gel electrophoresis. When circular plasmid DNA is conducted by electrophoresis, the fastest migration will be observed for the supercoiled form (Form I). If one strand is cleaved, the supercoil will relax to produce a slower-moving nicked circular form (Form II). If both strands are cleaved, a linear form (Form III) will be generated that migrates in between. The platinum complex was found to promote the cleavage of pUC19 plasmid DNA from supercoiled Form (I) to the nicked Form (II) by varying the concentration (100–400 *μ*M, figure not presented). The complexes can induce the obvious cleavage of the plasmid DNA at the concentration of 100 *μ*M.

**Figure 8:**
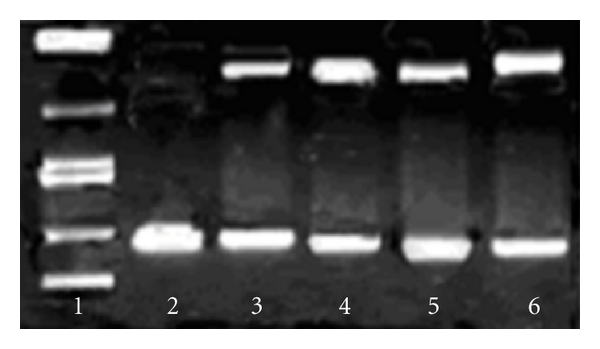
Cleavage of SC pUC19 DNA (50 *μ*M) by platinum complex (100 *μ*M) in the presence of H_2_O_2_ (100 *μ*M), in 10 mM Tris-HCl/1 mM EDTA buffer (pH 8.0). lane 1: DNA Marker; lane 2: DNA control; lane 3: DNA + complex; lane 4: DNA + complex 1 + H_2_O_2_; lane 5: DNA + complex + H_2_O_2_ + DMSO (4 *μ*L); lane 6: DNA + complex 1 + H_2_O_2_ + NaN_3_ (100 *μ*M).

One of the most interesting electrophoretic results of the complex takes place when experiment done in presence of H_2_O_2_, where the cleavage of the supercoiled DNA Form (I) into nicked DNA Form (II) take place more than complex alone. The mechanism of pUC19 DNA cleavage by our complex was then in the presence of studied by implementing various inhibiting reagents. The effect of reactive oxygen species on this process was tested with standard hydroxyl radical scavenger (DMSO) and singlet oxygen scavenger (NaN_3_). DMSO (lane 5 in Figure 8) remarkably inhibited the DNA breakage (36–40%) induced by the complexes (100 *μ*M). Interestingly, the singlet oxygen scavenger NaN_3_ failed to protect the DNA from the platinum complex induced cleavage almost (lanes 6 in Figure 8), which suggests that singlet oxygen does not play an important role in the cleavage mechanism pathway. In summary, these results indicate that the cleavage reaction involves hydroxyl radicals, that is, a Fenton type reaction may leads to the formation of these oxygen active species which finally cleave the DNA.

## 4. Conclusions

We recently investigated the interaction of PtCl_2_(NN) complex (NN = 4,7-dimethyl-1,10 phenanthroline) [49], [PtCl_2_(DIP)] [50] and [PtCl_2_(LL)] in which LL = N,N- Dimethyl-trimethylenediamine [51] with CT-DNA. The addition of solvents other than water, though, ensure solubility of those complexes, increases the hydrophobicity of the bulk solvent, which in turn decreases the DNA binding ability of the complexes. In the present study this difficulty has been overcome by using a water soluble complex, [Pt(DIP)LL)]^+2^ such a ligand modification would also provide us an opportunity to obtain addition structural insight into the binding event. In this study, we have synthesized a new Pt(II) complex, [Pt(DIP)(LL)](NO_3_)_2_ (in which DIP is 4,7-diphenyl-1,10-phenanthroline and LL is the aliphatic dinitrogen ligand, N,N- Dimethyl-trimethylenediamine) which exhibits high binding affinity to CT-DNA and the following results supported the fact that the complex can bound to CT-DNA by the mode of intercalation.

1. In absorption spectrum, the absorption intensity of the complex increased (hyperchromism) evidently after the addition of DNA, which indicated the interactions between DNA and the complex. The intrinsic binding constant (*K*_*b*_ = 6.6 × 10^4^ M^−1^) is roughly comparable to other intercalators [29]. Interestingly, the *K*_*b*_ value obtained for our complex is higher than that of the other platinum complex [PtCl_2_(NN)] (*K*_*b*_ = 6.35 × 10^4^) [49]. Therefore, the binding constant indicates that our complex can bind strongly with DNA.
2. The relative viscosity of DNA increases with increase in the concentration of Pt (II) complex which is ascribed to the intercalative binding mode of the complex because this could cause the effective length of the DNA to increase [38, 39].
3. Fluorescence studied results show that the probable quenching mechanism of Pt(II) complex formation by DNA is a dynamic quenching procedure, because the *K*_sv_ has been increased by temperature rising [49]. Thermodynamic studies showed that Δ*H* < 0 and Δ*S* < 0. Therefore, van der Waals interactions or hydrogen bonds are the main forces in the binding of the investigated Pt(II) complex to CT-DNA, and the mode of binding is intercalation.
4. Circular dichroism results showed deep conformational changes of CT-DNA double helix following the interaction with the complex.
5. The platinum complex was found to promote the cleavage of pUC19 plasmid DNA from supercoiled Form (I) to the nicked Form (II) by varying the concentration (100–400 *μ*M, figure not presented). The complexes can induce the obvious cleavage of the plasmid DNA at the concentration of 100 *μ*M.

## Figures and Tables

**Table 1:** ^1^H NMR chemical shifts of 4,7-diphenyl-1,10-phenanthroline (DIP) and *N,N*-dimethyltrimethylenediamine ligand (LL) and [Pt(DIP)(LL)](NO_3_)_2_ complex.

Protons	Ph	H^1′^	H^2^′	H^3^′	^1^CH_2_	^2^CH_2_	^3^CH_2_	NH_2_	Me
DIP	7.8	9.7	8.2						
LL					3.23	2.01	2.52	2.3	2.68
*[Pt(DIP)(LL)]^2+^	7.48, 7.6	9.58, 9.61	8.07, 8.11	8.01, 8.03	3.03	1.86	2.46	2.16	2.41

**Table 2:** The quenching constants of Pt (II) complex by CT DNA at different temperatures ranging from 0–5 × 10^−5 ^mol L^−1^ of CT DNA.

T (K)	*R* ^2^	*K*_sv_ (Lmol^−1^) × 10^3^	*K*_*q*_ (Lmol^−1^) × 10^11^
283	0.958	13.615	13.615
288	0.997	17.268	17.268
310	0.992	23.126	23.126

**Table 3:** Binding constants (*K*_*f*_), number of binding sites (*n*), and thermodynamic parameters of the Pt (II)-DNA system.

*T*(K)	*n*	Log *K*_*f*_	*K*_*f*_	*R* ^2^	Δ*G* ^0^ (kJ mol^−1^)	Δ*H* ^0^ (kJ mol^−1^)	Δ*S* ^0^ (J mol^-1 ^K^°−1^)
283	1.74	7.289	1.9 × 10^7^	0.94	−186.42	−111.79	−263.66
288	1.421	5.965	9.22 × 10^5^	0.994	−187.72	−111.79	−263.66
310	1.191	5.162	14.5 × 10^4^	0.987	−193.52	−111.79	−263.66

## References

[1] Garnuszek P, Liciánska I, Skierski JS (2002). Biological investigation of the platinum(II)-[∗I]iodohistamine complexes of potential synergistic anti-cancer activity. *Nuclear Medicine and Biology*.

[2] Loehrer PJ, Williams SD, Einhorn LH (1988). Testicular cancer: the quest continues. *Journal of the National Cancer Institute*.

[3] Keder A, Cohen ME, Freeman AI (1978). Peripheral neuropathy as a complication of cisdichlorodiammineplatinum
(II) treatment: a case report. *Cancer Treatment Reports*.

[4] Wong E, Giandomenico M (1999). Current status of platinum-based antitumor drugs. *Chemical Reviews*.

[5] Jakupec MA, Galanski M, Keppler BK (2003). Tumour-inhibiting platinum complexes—state of the art and future perspectives. *Reviews of Physiology, Biochemistry & Pharmacology*.

[6] De Almeida MV, Fontes APS, Berg RN, César ET, Felício EDCA, De Souza Filho JD (2002). Synthesis of platinum complexes from N-benzyl-1,3-propanediamine derivatives, potential antineoplastic agents. *Molecules*.

[7] Mudasir, Yoshioka N, Inoue H (1999). DNA binding of iron(II) mixed-ligand complexes containing 1,10-phenanthroline and 4,7-diphenyl-1,10-phenanthroline. *Journal of Inorganic Biochemistry*.

[8] Metcalfe C, Thomas JA (2003). Kinetically inert transition metal complexes that reversibly bind to DNA. *Chemical Society Reviews*.

[9] Kirti K, Patel KK, Edward A (2002). Aryl substituted ruthenium bis-terpyridine complexes: intercalation and groove binding with DNA. *Journal of Inorganic Biochemistry*.

[10] Pyle AM, Rehmann JP, Meshoyrer R, Kumar CV, Turro NJ, Barton JK (1989). Mixed-ligand complexes of ruthenium(II): factors governing binding to DNA. *Journal of the American Chemical Society*.

[11] Moulding VH, Miskowski VM (1991). The effect of linear chain structure on the electronic structure of Pt(II) diimine complexes. *Coordination Chemistry Reviews*.

[12] Momeni BZ, Hamzeh S, Hosseini SS, Rominger F (2007). Hydrogen-halide versus alkyl-halide oxidative addition in dimethyl platinum(II) complexes: crystal structure of [PtBr_2_(bpy)]. *Inorganica Chimica Acta*.

[13] Friaza GG, Botello AF, Perez JM, Prieto MJ, Moreno V (2006). Synthesis and characterization of palladium(II) and platinum(II) complexes with Schiff bases derivatives of 2-pyridincarboxyaldehyde. Study of their interaction with DNA. *Journal of Inorganic Biochemistry*.

[14] Conrad ML, Enman JE, Scales SJ (2005). Synthesis, characterization, and cytotoxicities of platinum(II) complexes bearing pyridinecarboxaldimines containing bulky aromatic groups. *Inorganica Chimica Acta*.

[15] Kennedy SD, Bryant RG (1986). Manganese-deoxyribonucleic acid binding modes. Nuclear magnetic relaxation dispersion results. *Biophysical Journal*.

[16]  Hodges KD, Rund JV (I975). Oxidative addition of halogens and pseudohalogens to dihalo(1,10-phenanthroline)platinum(II). *Inorganic Chemistry*.

[17] Wolfe A, Shimer GH, Meehan T (1987). Polycyclic aromatic hydrocarbons physically intercalate into duplex regions of denatured DNA. *Biochemistry*.

[18] Bloomfield VA, Crothers DM, Tioco I (1974). *Physical Chemistry of Nucleic Acids*.

[19] Liu CL, Zhou JY, Li QX, Wang LJ, Liao ZR, Xu HB (1999). DNA damage by copper(II) complexes: coordination-structural dependence of reactivities. *Journal of Inorganic Biochemistry*.

[20] Xiao Y-N, Zhan C-X (2002). Studies on the interaction of DNA and water-soluble polymeric Schiff base-nickel complexes. *Journal of Applied Polymer Science*.

[21] Satyanarayana S, Dabrowiak JC, Chaires JB (1993). Tris(phenanthroline)ruthenium(II) enantiomer interactions with DNA: mode and specificity of binding. *Biochemistry*.

[22] Waring MJ (1965). Complex formation between ethidium bromide and nucleic acids. *Journal of Molecular Biology*.

[23] Baba Y, Beathy CL, Kagemoto A, Gebelien C (1982). *Biological Activity of Polymers*.

[24] Vijayalakshmi R, Kanthimathi M, Subramanian V, Nair BU (2000). Interaction of DNA with [Cr(Schiff base)(H_2_O)_2_]ClO_4_. *Biochimica et Biophysica Acta*.

[25] Eriksson M, Leijon M, Hiort C, Nordén B, Gräslund A (1992). Minor groove binding of [Ru(phen)_3_]^2+^ to [d(CGCGATCGCG)]_2_ evidenced by two-dimensional nuclear magnetic resonance spectroscopy. *Journal of the American Chemical Society*.

[26] Silvestri A, Barone C, Ruisi C, Anselmo D, Riela S, Turco liveri V (2007). The interaction of native DNA with Zn(II) and Cu(II) complexes of 5-triethyl ammonium methyl salicylidene orto-phenylendiimine. *Journal of Inorganic Biochemistry*.

[27] Brodie CR, Collins JG, Aldrich-Wright JR (2004). DNA binding and biological activity of some platinum(II) intercalating compounds containing methyl-substituted 1,10-phenanthrolines. *Dalton Transactions*.

[28] Maheswari PU, Palaniandavar M (2004). DNA binding and cleavage activity of [Ru(NH_3_)_4_(diimine)]Cl_2_ complexes. *Inorganica Chimica Acta*.

[29] Maheswari PU, Rajendiran V, Palaniandavar M, Parthasarathi R, Subramanian V (2005). Enantiopreferential DNA binding: [{(5,6−dmp)_2_Ru}_2_(*μ*−bpm)]^4+^ induces a B-to-Z conformational change on DNA. *Bulletin of the Chemical Society of Japan*.

[30] Sigma DS, Mazuder A, Pemn DM (1993). Chemical nucleases. *Chemical Reviews*.

[31] Jin L, Yang P (1997). Synthesis and DNA binding studies of cobalt (III) mixed-polypyridyl complex. *Journal of Inorganic Biochemistry*.

[32] Liu F, Meadows KA, McMillin DR (1993). DNA-binding studies of Cu(bcp)^2+^ and Cu(dmp)^2+^: DNA elongation without intercalation of Cu(bcp)^2+^. *Journal of the American Chemical Society*.

[33] Shi S, Liu J, Li J (2006). Synthesis, characterization and DNA-binding of novel chiral complexes Δ- and Λ-[Ru(bpy)_2_L]^2+^ (L = *o*-mopip and *p*-mopip). *Journal of Inorganic Biochemistry*.

[34] Li FH, Zhao GH, Wu HX (2006). Synthesis, characterization and biological activity of lanthanum(III) complexes containing 2-methylene-1,10-phenanthroline units bridged by aliphatic diamines. *Journal of Inorganic Biochemistry*.

[35] Satyanarayana S, Dabrowiak JC, Chaires JB (1992). Neither Δ- nor Λ-tris(phenanthroline)ruthenium(II) binds to DNA by classical intercalation. *Biochemistry*.

[36] Eftink MR, Ghiron CA (1976). Fluorescence quenching of indole and model micelle systems. *Journal of Physical Chemistry*.

[37] Eftink MR, Ghiron CA (1981). Fluorescence quenching studies with proteins. *Analytical Biochemistry*.

[38] Peng B, Chao H, Sun B, Li H, Cao F, Ji L-N (2007). Synthesis, DNA-binding and photocleavage studies of cobalt(III) mixed-polypyridyl complexes: [Co(phen)_2_(dpta)]^3+^ and [Co(phen)^2^(amtp)]^3+^. *Journal of Inorganic Biochemistry*.

[39] Vaidyanatha VC, Nair BU (2003). Photooxidation of DNA by a cobalt(II) tridentate complex. *Journal of Inorganic Biochemistry*.

[40] Krisch-de Mesmaeker A, Orellana C, Barton JK, Turro NJ (1990). Ligand-dependent interaction of ruthenium(II) polypyridyl complexes with DNA probed by emission spectroscopy. *Photochemistry and Photobiology*.

[41] Cui FL, Fan J, Li JP, Hu. ZD (2004). Interactions between 1-benzoyl-4-*p*-chlorophenyl thiosemicarbazide and serum albumin: investigation by fluorescence spectroscopy. *Bioorganic & Medicinal Chemistry*.

[42] Kashanian S, Gholivand MB, Ahmadi F, Taravati A, Colagar AH (2007). DNA interaction with Al-*N*, *N*′-bis(salicylidene)2, 2′-phenylendiamine complex. *Spectrochimica Acta Part A*.

[43] Jiang M, Xie MX, Zheng D, Liu Y, Li XY, Chen X (2004). Spectroscopic studies on the interaction of cinnamic acid and its hydroxyl derivatives with human serum albumin. *Journal of Molecular Structure*.

[44] Ross PD, Subramanian S (1981). Thermodynamics of protein association reactions: forces contributing to stability. *Biochemistry*.

[45] Zhang G, Que Q, Pan J, Guo J (2008). Study of the interaction between icariin and human serum albumin by fluorescence spectroscopy. *Journal of Molecular Structure*.

[46] Naik DB, Moorthy PN, Priyadarsini KI (1990). Nonradiative energy transfer from 7-amino coumarin dyes to thiazine dyes in methanolic solutions. *Chemical Physics Letters*.

[47] Kashanian S, Askari S, Ahmadi F, Omidfar K, Ghobadi S, Tarighat FA (2008). In vitro study of DNA interaction with clodinafop-propargyl herbicide. *DNA and Cell Biology*.

[48] Ivanov VI, Minchenkova LE, Schyolkina AK, Poletayev AI (1973). Different conformations of double-stranded nucleic acid in solution as revealed by circular dichroism. *Biopolymers*.

[49] Shahabadi N, Kashanian S, Purfoulad M (2009). DNA interaction studies of a platinum(II) complex, PtCl_2_(NN) (NN = 4, 7-dimethyl-1,10-phenanthroline), using different instrumental methods. *Spectrochimica Acta Part A*.

[50] Shahabadi N, Kashanian S, Fatahi A Identification of binding mode of a platinum (II) complex, PtCl_2_(DIP) (DIP = 4, 7-diphenyl-1,10-phenanthroline) and calf thymus DNA.

[51] Shahabadi N, Kashanian S, Shalmashi K, Roshanfekr H (2009). DNA interaction with PtCl_2_(LL) (LL = Chelating diamine ligand: *N*, *N*-dimethyltrimethylendiamine) complex. *Applied Biochemistry and Biotechnology*.

